# Enhanced information processing in the human neocortex: cellular mechanisms and translational perspectives

**DOI:** 10.3389/fnsyn.2026.1770193

**Published:** 2026-02-13

**Authors:** Manuela Tore, Laura Monni, Alessio Di Clemente, Michele Giugliano

**Affiliations:** 1 Department of Biomedical, Metabolic and Neural Sciences, University of Modena and Reggio Emilia, Modena, Italy; 2 Neuroscience Area, International School for Advanced Studies (SISSA), Trieste, Italy; 3 National Interuniversity Consortium of Materials Science and Technology, Firenze, Italy

**Keywords:** action potential kinetics, AP dynamics, dendritic trees, human neurons, ion channel kinetics, layer 2/3 pyramidal cells, membrane capacitance, perisomatic branching

## Abstract

Understanding the sophisticated cognitive abilities of the human brain requires understanding its cellular and synaptic components. While rodent studies provide foundational knowledge, recent research using freshly resected human neocortical and hippocampal tissue has revealed unanticipated distinctive cellular characteristics. These properties, identified through *in vitro* electrophysiology, anatomical reconstructions, and computational modeling, have profound implications for physiological processes and modulatory responses. Here we highlight and review a selection of key unique features of human neurons. Human layer 2/3 pyramidal cells exhibit exceptionally low specific membrane capacitance and distinctive ion channel kinetics. Moreover, human pyramidal-to-pyramidal connections display species-specific synaptic dynamics, recovering from short-term depression much faster than in rodents. We also highlight that human pyramidal neurons exhibit more elaborate dendritic trees, particularly perisomatic branching, and faster, more stable Action Potentials (AP) dynamics. Interestingly, these features allow higher-bandwidth information transfer, reflecting enhanced computational power. All these cell-level differences directly impact how circuits process information and respond to pharmacological interventions. Increasingly, drugs targeting ion channels or synaptic mechanisms are used but often display different efficacy or kinetics in human neurons compared to rodents, reflecting underlying biophysical disparities. Consequently, leveraging human brain tissue is key as it allows for the identification of human-specific drug targets and a more accurate understanding of disease mechanisms. This review highlights these crucial cellular distinctions and underscores the importance of exploiting resected human brain tissue for advancing central nervous system therapeutics.

## Introduction

Every biological species exhibits distinctive traits and strategies shaped by evolutionary processes to enhance its fitness. *Homo sapiens* stands out for its elaborate cognitive capabilities and the associated behavioral complexity. For example, humans developed written and spoken symbolic languages, technologies far more complex than those observed in any other species and they are able to produce and appreciate art (Laland and Seed, 2021). Although not necessarily “unique” to humans, these behaviors differ from those observed in other species quantitatively and in their ecological relevance.

To date, it is not fully clear where this cognitive and behavioral complexity originates from. At the macroscale, one of the major differences is the expansion of the neocortex. Humans have the largest expansion among primates with the cerebral cortex occupying 75–82% of the whole brain volume (Jardim-Messeder et al., 2017). Nevertheless, brain size and number of neurons are not that different between humans and other species. The human brain is not the first in relative brain size (brain-to-body index), in absolute brain size, or gyrification (Hofman, 1988; Hofman, 1985). In addition, neuronal density in cortical areas is lower in the human brain than in other species (DeFelipe, 2011), and despite its larger relative size, the human cortex contains roughly the same proportion of neurons (20–25% of the total) found in other mammals. These features highlight that brain function is shaped by multiscale processes, not only by cortical size or other macroscopic properties. Therefore, our cognitive specialization could be rooted deeper in the characteristics of its basic elements, i.e., single neurons (Galakhova et al., 2022).

**Figure 1:**
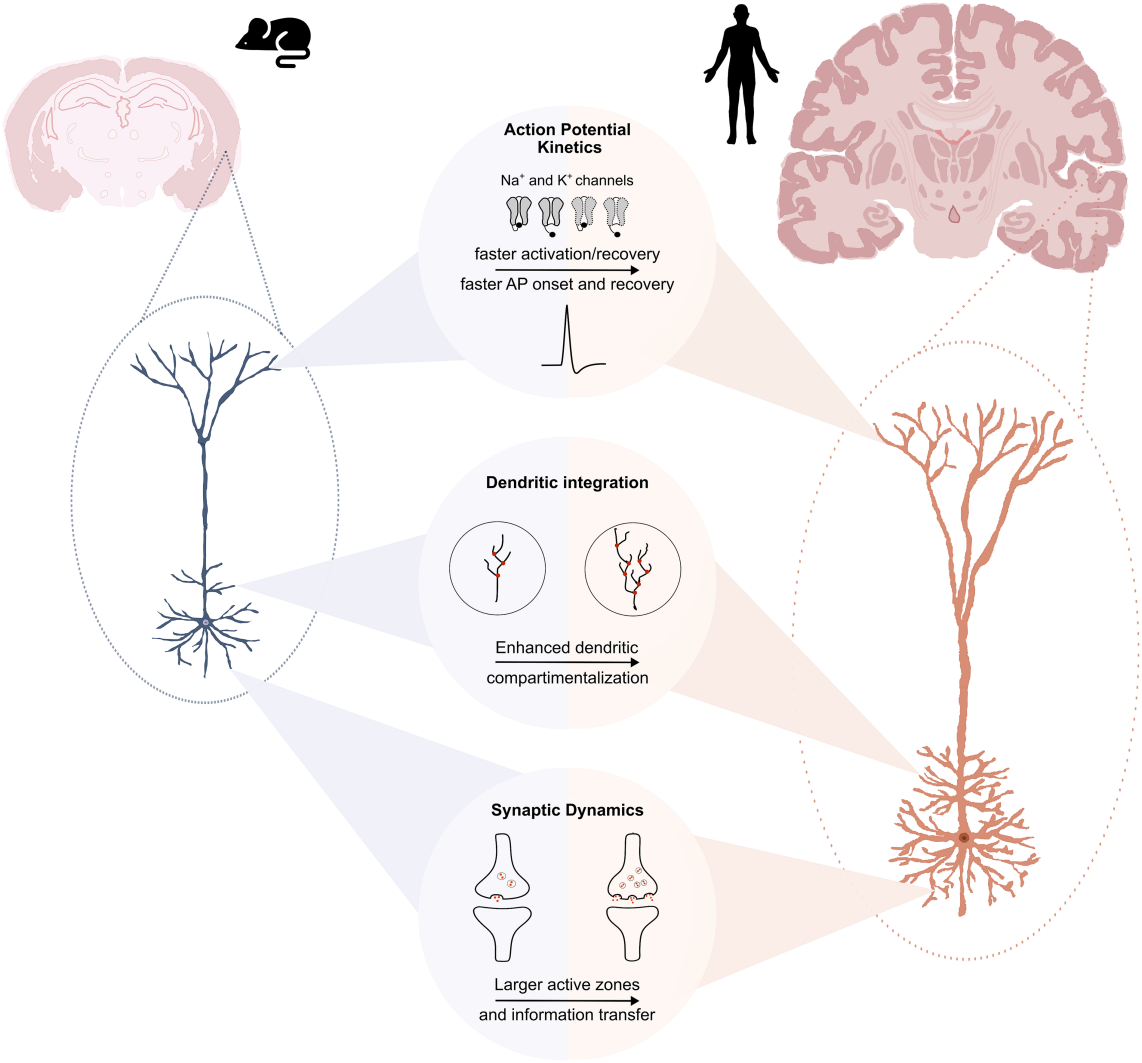
Unique cellular properties of human pyramidal neurons. **Top panel:** Voltage-dependent Na^+^ and K^+^ channels in human pyramidal neurons operate at more depolarized voltage ranges compared to their mouse counterparts. As a result, Na^+^ channels recover faster from inactivation, while K^+^ channels activate rapidly at lower membrane potentials. Compared to rodents, human pyramidal neurons display faster action potential (AP) onset and more stable recovery during repetitive firing, supporting reliable high-frequency signaling and high-fidelity encoding of temporally structured inputs. **Middle panel:** Human pyramidal neurons are not simply enlarged versions of mouse neurons. They exhibit substantially greater total dendritic length (TDL), dendrites are positioned closer to the soma and with larger diameters, and they show a higher number of dendritic branches—particularly within the basal dendritic arbor. These morphological specializations increase dendritic compartmentalization, allowing human neurons to operate with more functionally segregated dendritic domains. This property supports parallel integration of synaptic inputs and localized nonlinear processing, ultimately expanding the computational repertoire of an individual cell. **Bottom panel:** Human synapses contain larger active zones with a higher number of functional release sites. These properties, together with a faster recovery from short-term depression, help prevent vesicle depletion during high-frequency stimulation, support multivesicular release, and ultimately enable a larger dynamic range of information transfer with increased reliability during sustained or temporally structured inputs compared to rodent synapses. These features make neurons sensitive to subtle presynaptic activity variations and enhance their ability to encode information accurately.

Indeed, differences between human and rodent neurons are increasingly recognized as crucial for understanding species-specific cortical computation and for improving translational neuroscience (Figure 1). To reliably identify human-specific neuronal properties, it is essential to find experimental models that closely capture the molecular, functional, and physiological features of the human brain. Acute slices prepared from freshly resected tissue offer this level of fidelity, preserving native cytoarchitecture, gene expression profiles, and functional activity. Although these samples are typically obtained from patients undergoing surgery for drug-resistant epilepsy or brain tumors, they retain key physiological characteristics making them a powerful model for studying human neuronal function (Bak et al., 2024; Chameh et al., 2023; Kraus et al., 2020; Ravi et al., 2019; Schwarz et al., 2019; Ting et al., 2018). Animal models are fundamental for basic mechanistic and translational studies, yet they also contribute to the well-recognized gap in translational neuroscience, as drugs targeting ion channels, synapses, or neurotransmitter systems, often fail to reproduce their predicted efficacy in humans. This challenge has been increasingly highlighted by both academia and industry, contributing to the broader crisis in preclinical-to-clinical translatability. Therefore, using human tissue models accurately reflecting human pathophysiology may help to narrow this “valley of death” and improve the success of central nervous system drug development (Akhtar, 2015; Ledri et al., 2015; Seyhan, 2019).

In this review, we summarize recent findings concerning said differences, with a particular emphasis on single-cell electrophysiology studies performed on freshly resected human brain samples. Human-specific features identified in this context can be grouped into three domains: (1) Action potential (AP) kinetics, (2) dendritic integration, (3) synaptic dynamics. Each domain is considered separately, and throughout the review we highlight how their interplay contributes to distinctive computational and information-processing properties. Notably, these peculiar properties may also influence microcircuit activity and response to therapeutic treatments, providing a possible ground to explain, at least partially, why most of the drugs identified on animal models in the preclinical phase fail to translate to humans.

## Action potential kinetics

Action potential (AP) kinetics are largely determined by voltage-gated ion channels. Their properties and relative expression in different cell compartments shape the AP and strongly contribute to neurons’ intrinsic excitability and electrical phenotype. Consequently, differences in their activation and inactivation kinetics influence a neuron’s ability to fire at different voltages and across different firing frequencies (i.e., the f-I curve) (Hille, 1978).

**Figure 2:**
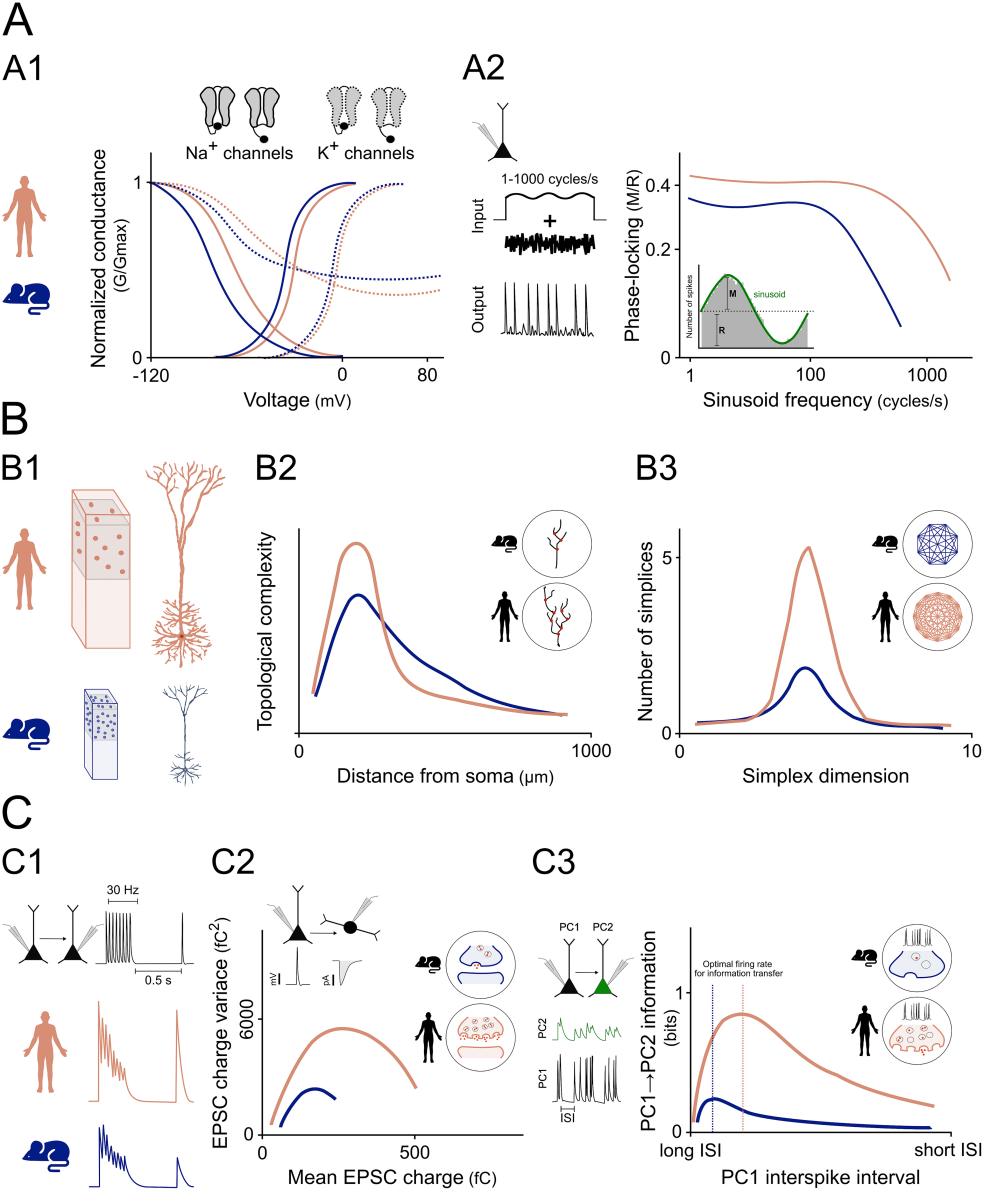
Human-specific cellular and synaptic features support enhanced information processing. **(A)** Ion channel kinetics and input–output encoding. **(A1)** Steady-state activation and inactivation curves for Na^+^ and K^+^ channels are shifted to more depolarized voltages in human neurons. **(A2)** Schematic of a pyramidal neuron receiving a subthreshold sinusoidal input (1–1,000 cycles/s) with randomly fluctuating (noise) current component, resulting in a train of action potentials. Conceptual plot showing phase-locking strength (modulation/mean firing rate, M/R) as a function of input frequency. Human neurons maintain stronger phase-locking across a broader frequency range than mouse neurons, as indicated by a higher cut-off frequency. Inset: schematic of phase-locking quantification. Images adapted from Wilbers et al. (2023) and Testa-Silva et al. (2014) only for illustrative purposes. **(B)** Dendritic morphology, and network topology. **(B1)** Conceptual comparison of rodent vs. human cortical pyramidal neurons showing larger size, longer dendrites, increased branching, sparser distribution. **(B2)** Schematic of dendritic topological complexity as a function of distance from the soma, emphasizing increased perisomatic branching in human neurons. Inset: branch density comparison. **(B3)** Conceptual plot of network complexity illustrating a higher number of simplices across dimensions in human circuits. Simplices can be roughly defined as high-order topological shapes with minimum number of vertices in space, reflecting the minimal unit of collective interaction. Higher-dimensional simplices are associated with increased robustness in networks. Inset: schematic showing denser, higher-order connectivity enabled by increased dendritic complexity. Images adapted from Kanari et al. (2025) only for illustrative purposes. **(C)** Synaptic dynamics and AP kinetics. **(C1)** Paired recordings illustrating short-term synaptic depression during 30 Hz stimulation, followed by recovery after 0.5 s. Human synapses show faster recovery than rodent synapses. **(C2)** Conceptual EPSC variance–mean plot used in multiple probability fluctuation analysis of synaptic transmission reveals a higher number of functional release sites in human neurons. Inset: schematic of enhanced release site number. **(C3)** Paired recordings of synaptically connected pyramidal neurons (PC1 and PC2). PC1 fires Poisson-like spike trains with variable inter-spike intervals (ISI), while PC2 exhibits corresponding EPSPs (green). Conceptual plot illustrating information transfer (calculated by mutual information between peak EPSP amplitudes and presynaptic interspike intervals), showing that human synapses reach optimal information transmission at higher firing rates (shorter ISIs) compared to rodent synapses. Inset: Schematic comparison of presynaptic terminals showing that, at equivalent presynaptic firing frequencies, human synapses maintain higher vesicle availability and exhibit less vesicle depletion than rodent synapses. Images modified from Testa-Silva et al. (2014) and Molnár et al. (2016) only for illustrative purposes.

Wilbers et al. (2023) investigated the biophysical properties of voltage-gated Na^+^ and K^+^ currents in human and mouse cortical pyramidal neurons. They found that steady-state voltage-dependence curves of human currents were significantly right-shifted compared to mouse currents: Na^+^ activation and inactivation curves were shifted by 6 and 9 mV, respectively, while K^+^ activation and inactivation curves were shifted by 5 and 12 mV, respectively (Figure 2A).

Furthermore, human voltage-gated Na^+^ currents exhibited slower activation and inactivation time constants across all voltages, whereas human K^+^ currents activated faster at lower membrane potentials and showed similar inactivation time constants to rodents. Importantly, despite their slower activation/inactivation kinetics, Na^+^ currents recover from inactivation significantly faster (Wilbers et al., 2023).

On one side, these differences result in a larger overlap between Na^+^ and K^+^ currents, and thus in a larger excess influx of sodium ions during AP falling phase (data shown in supplementary material in Wilbers et al., 2023). Since restoring ion concentrations across the membrane requires ATP-dependent processes, the metabolic cost of firing an AP is expected to be higher in human neurons. On the other hand, slower inactivation and faster recovery from inactivation of Na^+^ channels increase the fraction of channels available after an AP has been fired (Wilbers et al., 2023).

Wilbers et al. (2023) proposed that these properties, and particularly the higher Na^+^ channel availability resulting from the faster recovery, support faster and more reliable AP firing and thus a faster input–output conversion. Accordingly, they showed that the AP relative rise speed after 200 AP at 70 Hz in human neurons was 0.75 of the first AP generated, whereas in rodents it dropped to 0.56 (Wilbers et al., 2023). An earlier study from Wang corroborates these findings, showing that human cortical neurons could generate APs at a maximal mean frequency of 338 Hz, whereas the maximal mean frequency of mouse pyramidal neurons was 215 Hz (Wang et al., 2016). Another critical aspect is the AP onset rapidity. Testa-Silva et al. (2014) quantified AP onset rapidity in rat and human cortical neurons, reporting higher values in the latter (Testa-Silva et al., 2014) (Figure 2C).

Faster and more stable AP kinetics enable more consistent modulation of AP firing on fine temporal scales, determining whether subthreshold membrane potential oscillations generated by synaptic activity are encoded into AP output (Ilin et al., 2013). This had been widely anticipated in theoretical studies (Ilin et al., 2013; Fourcaud-Trocmé et al., 2003) where explicit links were demonstrated between AP initiation properties and enhanced input–output information encoding as well as self-consistent oscillatory network regimes (Geisler et al., 2005). With faster AP onset, human cortical pyramidal neurons may therefore track subthreshold oscillations more reliably across a wider range of frequencies, resulting in an input–output transfer function with greater bandwidth and higher signal-to-noise ratio. Testa-Silva et al. (2014) tested this by delivering small sinusoidal inputs of different frequencies on top of a randomly fluctuating current in human and mouse neocortical pyramidal neurons. While the fluctuating current induced an irregular firing regime with low average rate (~10–15 Hz), the superimposed sinusoidal currents modulated instantaneous firing probability. Phase-locking of AP firing probability (quantified as modulation/mean firing rate, M/R) was stronger in human neurons across all frequencies (Figure 2A), with significant phase-locking up to 1,000 cycles/s. By contrast, mouse pyramidal neurons showed little or no significant modulation for input frequencies above ~100–200 cycles/s (Testa-Silva et al., 2014).

Cortical layers can display different specific properties, reflecting physiologically relevant aspect of human cortical diversity (Chameh et al., 2023). In contrast to human layer 5, where measurements from pyramidal neurons showed passive properties comparable to rodent ones (Brette, 2013), Eyal et al. (2016) reported that the specific membrane capacitance (Cm) of human layers 2/3 cortical neurons is approximately half the commonly accepted value (~0.5 μF/cm^2^ in humans vs. ~1 μF/cm^2^ in rodents). If confirmed, such a reduction in Cm may substantially contribute to the faster input–output transformation observed in human layers 2/3 neurons. A lower Cm decreases the membrane time constant and thus the time-scale of synaptic inputs integration, allowing membrane potentials to change more rapidly. In Rall’s cable equation, a lower Cm accounts for higher spatial conduction velocity for transient dendritic stimuli (Rinzel and Rall, 1974). Moreover, larger excitatory postsynaptic potentials (EPSPs), improved dendro-somatic transfer, and faster axonal propagation and AP onset rapidity were shown in realistic multicompartmental models of pyramidal neurons when decreasing the Cm value (Eyal et al., 2016).

These electrophysiological distinctions are not only relevant to basic signaling but may also correlate with higher cognitive functions. In fact, recent studies have suggested a correlation between ion channel properties and intelligence quotient (IQ) across species (Goriounova et al., 2018).

These species-specific differences may be particularly relevant for understanding human neuronal excitability and network dynamics in pathologies such as epilepsy, where deregulated neuronal activity is key. While their direct contribution to drug responsiveness remains to be determined, they highlight the importance of studying human neurons when investigating disease mechanisms and potential therapeutic interventions.

## Dendritic integration

Human pyramidal neurons, in both cortical layers 2/3 and hippocampus are sparser than their rodent counterparts (Kanari et al., 2025; Watson et al., 2025). Nevertheless, human cortical circuits exhibit greater structural complexity, a feature widely hypothesized to arise from an increased morphological complexity of the dendritic tree (Kanari et al., 2025; Watson et al., 2025; Eyal et al., 2014) (Figure 2B).

Interestingly, a modeling study from Eyal and collaborators reported that a large size of dendritic tree could increase AP onset rapidity by imposing a larger impedance on the axon initial segment (AIS), where the AP is initiated, via electrotonic interaction (Eyal et al., 2014; Brette, 2013; Zhang et al., 2022). This offers an additional mechanistic explanation to the faster AP kinetics reported for human neurons.

The presence of this larger dendritic tree in human neurons is supported by studies comparing length and structural complexity of human and rodent dendritic arborizations.

Kanari and colleagues analyzed morphologies from several studies and found that neocortical layer 2/3 human pyramidal neurons exhibit almost double the Total Dendritic Length (TDL) of their rodent counterparts (11.6 ± 5.5 mm vs. 5.5 ± 2.2 mm, respectively) (Kanari et al., 2025). Similar findings were also reported for CA3 and CA1 areas of the hippocampus. TDL measurements indicate substantial differences between species: in CA3, TDL reaches 12.5 ± 0.4 mm in humans, 7.5 ± 0.2 mm in rats, and 6.5 ± 0.2 mm in mice (Watson et al., 2025). In CA1 pyramidal neurons TDL was 18.6 ± 3.9 mm in humans, 12.9 ± 1.5 mm in rats, and 3.7 ± 0.6 mm in mice (Mertens et al., 2024). Human pyramidal neurons’ dendritic arborizations are not only longer but also substantially more elaborate than rodent ones, with more branches in both, apical (54 ± 21 in humans vs. 36 ± 11 in mice) and basal (61 ± 34 in humans vs. 46 ± 19 in mice) dendrites (Watson et al., 2025).

Kanari et al. (2025) reported another fundamental difference in the branching pattern of human vs. mouse neocortical pyramidal neurons. Specifically, human dendrites exhibit initial branching points that lie closer to the soma and extend to longer radial distances. This results in a distinct topological profile characterized by longer and more densely packed dendritic branches within the proximal domain (200–500 μm from the soma) (Figure 2B). Interestingly, this topological profile appears to generalize across multiple brain regions and may represent a characteristic feature of human pyramidal neurons (Watson et al., 2025; Benavides-Piccione et al., 2020).

Additionally, perisomatic dendrites are larger in humans, with proximal segments exhibiting diameters nearly twice those of mice, whereas distal dendritic diameters are comparable between the two species (Wilbers et al., 2023).

Taken together, the substantially longer dendrites and the larger proximal dendritic diameters promotes greater attenuation of synaptic signals as they propagate toward the soma. These features effectively increase the electrotonic length of the neuron, rendering distal dendrites more electrically remote and thereby enhancing dendritic compartmentalization, with important consequences for synaptic integration (Eyal et al., 2014).

Another relevant difference between human and rodent dendrites concerns the distribution of hyperpolarization-activated cyclic nucleotide-gated (HCN) channels, which conducts both potassium and sodium ions. In contrast to rodents, somatic and dendritic HCN conductance in human neurons does not scale with neuron size, resulting in lower channel density, higher dendritic input resistance, and local modulation of dendritic excitability (Brette, 2013).

In this context, theoretical studies have suggested that human dendritic compartments can operate as relatively independent computational subunits, effectively acting as “logical gates” capable of parallel processing and nonlinear transformation of synaptic inputs, rather than functioning as simple linear summators at the soma (Häusser and Mel, 2003; London and Häusser, 2005; Jadi et al., 2014; Poirazi et al., 2003; Polsky et al., 2004; Tran-Van-Minh et al., 2015).

Furthermore, a higher dendritic topological complexity leads to an increased dendritic memory capacity, reflecting a neuron’s ability to store information in localized, independent synaptic compartments (Kanari et al., 2025; Goriounova et al., 2018; Watson et al., 2025; Poirazi and Mel, 2001). Lastly, the group of Kanari et al. (2025) estimated the number of appositions (i.e., contact points that can potentially be synapses) from data on cell densities and morphologies in human and mouse tissue. Assigning connections to a random selection of 50% of the appositions, they estimated network connectivity and evaluated its complexity. Their results suggest that the topological profiles of human neurons may give rise to highly interconnected and complex subnetworks. Indeed, the pyramidal-cell subnetworks exhibited rich connectivity patterns supporting the formation of higher-dimensional simplices (Kanari et al., 2025; Reimann et al., 2017) (Figure 2B). Such higher order structures might imply that these networks can process higher order correlations among groups of neurons rather than being limited to pairwise interactions.

All this considered, a single human pyramidal neuron could perform computations similar to a multilayered rodent network (Galakhova et al., 2022), leading to different network dynamics and possibly altered responses to drug treatments compared to animal models.

## Synaptic dynamics

Given the enormous density of synapses in the cortex, even the smallest change in their dynamics could dramatically affect the brain’s overall computational performance (Testa-Silva et al., 2014).

In contrast to rodents, human layer 2/3 pyramidal neurons’ monosynaptic connections showed only frequency-dependent depression, without potentiation, and recovered more rapidly: EPSP amplitudes returned to baseline within 0.5 s after 30 Hz stimulation, a phenomenon not observed in rodents’ synapses (Testa-Silva et al., 2014) (Figure 2C).

Beyond this, a key difference concerns the number of functional release sites per synaptic contact. In human layer 2/3 pyramidal-to-fast-spiking-interneuron synapses, paired recordings and fluctuation analysis estimated roughly four times more release sites per connection compared to rats (Molnár et al., 2016) (Figure 2C). Electron microscopy (EM) measurements demonstrated that human presynaptic active zones (AZ) are larger (average ~0.077 μm^2^ vs. ~ 0.041 μm^2^ in rats) and possess about four docked vesicles per AZ, compared to only one in rats. Correspondingly, each AZ in the human cortex has been shown to contain ~6 functional release sites, whereas rat AZs contain ~1.6, reflecting a capacity for multivesicular release in humans (Molnár et al., 2016). Notably, AZs and postsynaptic densities of excitatory synaptic boutons in human neocortical layer 5 were comparable in size to those in rat layers 4 and 5. Nevertheless, human layer 5 synapses exhibit larger vesicle and ready releasable pools (RRP), approximately two- to three-fold bigger than in rats (Yakoubi et al., 2019). Although quantified in different cell classes, these findings converge on a consistent principle: human synapses contain larger vesicle pools and support stronger multivesicular output.

Together, these structural and functional specializations result in a stronger and more reliable excitatory drive, increased sensitivity to subtle changes in presynaptic activity, and consequently to larger synapses’ dynamic range and information transfer. The latter is defined as the amount of information a neuronal response conveys about its evoking stimulus and depends critically on synaptic efficiency and recovery (Borst and Theunissen, 1999). The larger RRP and the faster recovery from depression prevent synapses from becoming depleted during high-frequency discharges, enabling them to continue transmitting information reliably over a broader frequency range (Figure 2C). Accordingly, information-theoretic analyses by Testa-Silva and colleagues (Testa-Silva et al., 2014) estimated that peak information transfer at these synapses can be approximately four- to nine-fold greater in humans than in rodents. Moreover, if specific membrane capacitance is indeed lower in human neurons, this may further facilitate membrane potential deflections, enhancing synaptic efficacy and information transmission (Testa-Silva et al., 2014; Yakoubi et al., 2019). These synaptic specializations contribute to sub-millisecond spike-timing precision and more consistent temporal encoding in human neurons, enabling more precise transmission of downstream information compared to rodents (Testa-Silva et al., 2014).

Consistent with the stronger excitatory drive and the highly interconnected network, functional recordings from human neocortical tissue showed that a single AP in a layer 2/3 pyramidal neuron can trigger prolonged polysynaptic activity. This phenomenon was characterized by alternating excitatory and inhibitory events that recruited both glutamatergic and GABAergic neurons and propagated through the network for tens of milliseconds (Molnár et al., 2008).

## Conclusion

In this short review, we presented the main differences between human and rodent pyramidal neurons of the neocortex. We highlighted that human pyramidal neurons show distinct AP kinetics, enabling faster input–output conversion and generation of APs at significantly higher firing frequencies than their rodent counterparts. Additionally, human pyramidal neurons display more complex morphology and dendritic topology, leading to subnetwork with more nodes, greater compartmentalization, and enhanced computational capacity. Finally, we focused on how human neurons exhibit different synaptic dynamics. Larger AZs and RRPs allow single human synapses to perform multivesicular release, which increases the precision of downstream information transfer compared to rodent synapses. Taken together, these disparities - including debated differences in specific membrane capacitance - highlight that individual neurons in the human brain possess distinctive properties that cannot be fully inferred from rodent studies. If confirmed, features like a potentially lower specific membrane capacitance (Eyal et al., 2016) could have profound functional consequences, influencing signal processing, membrane potential deflections, and spike propagation. Even in the absence of full consensus (Goriounova et al., 2018; Beaulieu-Laroche et al., 2018; Gooch et al., 2022) these potential differences underscore the limitations of extrapolating data from rodents to humans and reinforce the necessity of studying electrophysiological properties directly in freshly resected tissue to accurately understand human brain physiology and pathology. Such investigations help clarify the features that distinguish human neurons from those of other species. At the same time, it is important to acknowledge that these samples originate from patients, often with neurological conditions, and therefore may not fully reflect healthy human brain tissue. However, it remains essential to disentangle the interactions between single-neuron and network activity by using advanced electrophysiology tools, such as high-density microelectrode arrays or multipatch setups, which allow a high-yield data collection with fewer experiments. In fact, only by directly studying the human brain we can fully understand its pathophysiology and thereby pave the way for more effective therapeutic approaches.
